# Enhanced thermoelectric performance of UV-curable silver (I) selenide-based composite for energy harvesting

**DOI:** 10.1038/s41598-021-96267-x

**Published:** 2021-08-17

**Authors:** Dabin Park, Seonmin Lee, Jooheon Kim

**Affiliations:** 1 School of Chemical Engineering & Materials Science, Chung-Ang University, Seoul, 06974 Republic of Korea; 2 Department of Advance Materials Engineering, Chung-Ang University, Anseong, 17546 Republic of Korea; 3 Department of Intelligent Energy and Industry, Graduate School, Chung-Ang University, Seoul, 06974 Republic of Korea

**Keywords:** Thermoelectric devices and materials, Chemical engineering

## Abstract

Thermoelectric (TE) composites, with photocured resin as the matrix and Ag_2_Se (AS) as the filler, are synthesized by a digital-light-processing (DLP) based 3D printer. The mixture of diurethane dimethacrylate (DUDMA) and isobornyl acrylate (IBOA) is used as a UV-curable resin because of their low viscosity and high miscibility. Scanning electron microscopy (FE-SEM) images confirm that the filler retains its shape and remains after the UV-curing process. After completing curing, the mechanical and thermoelectric properties of the composite with different AS contents were measured. The addition of the AS filler increases the thermoelectric properties of the cured resin. When the AS contents increase by 30 wt.%, the maximum power factor was obtained (~ 51.5 μW/m·K^2^ at room temperature). Additionally, due to the phonon scattering effect between the interfaces, the thermal conductivity of composite is lower than that of pristine photoresin. The maximum thermoelectric figure of merit (*ZT*) is ~ 0.12, which is achieved with 30 wt.% of AS at 300 K with the enhanced power factor and reduced thermal conductivity. This study presents a novel manufacturing method for a thermoelectric composite using 3D printing.

## Introduction

Thermoelectric (TE) is a phenomenon that can convert heat directly into electricity and vice versa, which is attractive because it generates electricity from waste heat energy and provides environmentally friendly cooling^1–5^. Given the many advantages, many studies have been done to investigate a wide range of applications in the medical, military, telecommunication, and industrial fields^6–9^. The performance of a thermoelectric device directly depends on the inherent conversion efficiency of the materials, *ZT* = *S*^2^*·σ·T/к*, where *S* is the Seebeck coefficient, *σ* is the electrical conductivity, *к* is the thermal conductivity*,* and *T* is the absolute temperature. Theoretically, to get a high figure of merit, TE materials must have low thermal conductivity and a high-power factor (*PF* = *S*^*2*^*·σ*). Therefore, many studies on TE materials have focused on the requirements of a significant increase in the power factor or a decrease in thermal conductivity.

Many efforts on achieving high ZT in recent years are nanostructuring the materials and, therefore, decreasing the thermal conductivity. To date, most TE materials are reported as inorganic materials such as conducting oxides (NaCo_2_O_4_, SrTiO_3_, and CaMnO_3_)^10–15^. Half-heuslers^16^, and semiconductor materials contain Te and Se alloys (Bi_2_Te_3_, SnSe, Cu_2_Te, and PbTe)^17–21^. However, these pristine inorganic-based TE materials are brittle and rigid, making it difficult to manufacture flexible or wearable TE devices.

Therefore, studies have recently been conducted to maintain the high TE properties of inorganic materials by producing composites and diversifying their shapes. Among the inorganic materials used in these TE composites, silver selenide (Ag_2_Se) is an N-type semiconducting materials that is widely used as TE materials because of its high electrical conductivity and low thermal conductivity at room temperature. For example, Jiang et al.^22^ fabricated a flexible TE film with a PVP/Ag_2_Se composite and achieved an outstanding power factor with ~ 1910 μW/m·K^2^ at room temperature. Zhou et al.^23^ fabricated PVDF/Ag_2_Se nanocomposites with high Ag_2_Se loading and the maximum power factor of the flexible film is ~ 181 μW/m·K^2^.

Additive manufacturing is a technology that can print a variety of composite shapes with low material waste and a short production time^24,25^. Due to its unique characteristics, various studies using additive manufacturing are rapidly increasing. 3D printing uses a variety of methods, such as inkjet printing, fused deposition modeling (FDM), selective laser sintering (SLS), stereolithography apparatus (SLA), and digital light processing^26,27^. Yang et al.^28^ fabricated a BiSbTe-based TE generator using an inkjet 3D printing technique and reported a high *ZT* with ~ 1.1. Wang et al.^29^ achieved a maximum power factor of 11.3 μW/m·K^2^ with a wire-like PLA/MWCNT/BiSbTe composite, which was printed with 3D printing. DLP is considered a promising additive manufacturing technology among the many types of 3D printers due to its unique characteristic. DLP can print many samples within a single printing session. Also, by making photocured resins containing various fillers, composites for specific purposes can be printed.

In this study, we suggest a strategy to fabricate various shapes of thermoelectric materials using the 3D printer of the DLP method. The UV-curable resin used in this study is composed of diurethane dimethacrylate (DUDMA) and isobornyl acrylate (IBOA), and silver (I) selenide (Ag_2_Se) is chosen as the TE fillers. We designed a CAD design with various structures, and UV-curable resin with various Ag_2_Se contents is printed with this design using a 3D printer. After that, the mechanical and TE properties (*S, σ, κ, PF and ZT*) of our 3D-printed composite are measured. To the best of our knowledge, fabricating TE materials using a DLP-type 3D printer is novel and significant for manufacturing various shapes of TE devices.

## Results and discussion

**Figure 1:**
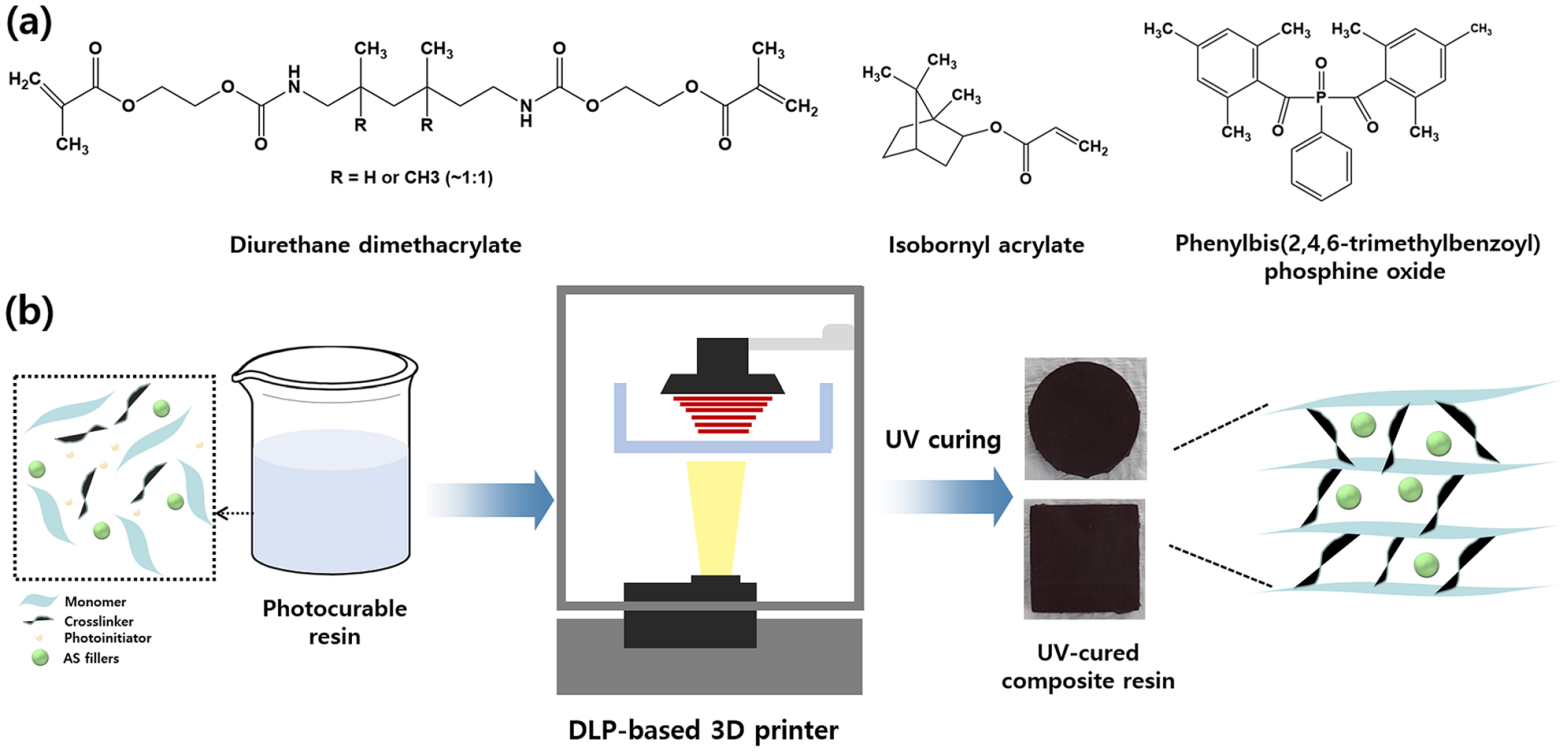
(**a**) Chemical structures of formulated photoresin. (**b**) Schematic illustration of composite sample preparation.

The composite sample preparation process is illustrated in Fig. 1. The formulation of the photoresin used in this study consists of a urethane monomer of DUDMA, a crosslinker of IBOA, and a photoinitiator of BAPO. At first, photoresin with varied Ag_2_Se contents (0, 10, 20, and 30 wt.%) are fabricated. The composition of each composite resin is listed in Table S1. It has been shown that when the AS content is more than 30 wt.%, the dispersion between the photoresin and AS fillers decreases, and the composite resin was not well-formed. After synthesizing the photoresin containing Ag_2_Se, all composite resins were printed with a DLP-based 3D printer. The exposure time was set at 5 s, and each layer of composite sample was set at 0.05 mm. The computer-aided design (CAD) model file was entered into the software of an ASIGA 3D printer. Then the printing proceeds through the composite resin in the tank. Composite resins can be cured into a variety of shapes and sizes. In Fig. S1, the various CAD designs and the 3D printed samples are illustrated, which can confirm the successful printing process. Finally, the cured composites were thermally annealed in N_2_ gas in a tubular furnace at 123 °C, for 5 h.

**Figure 2:**
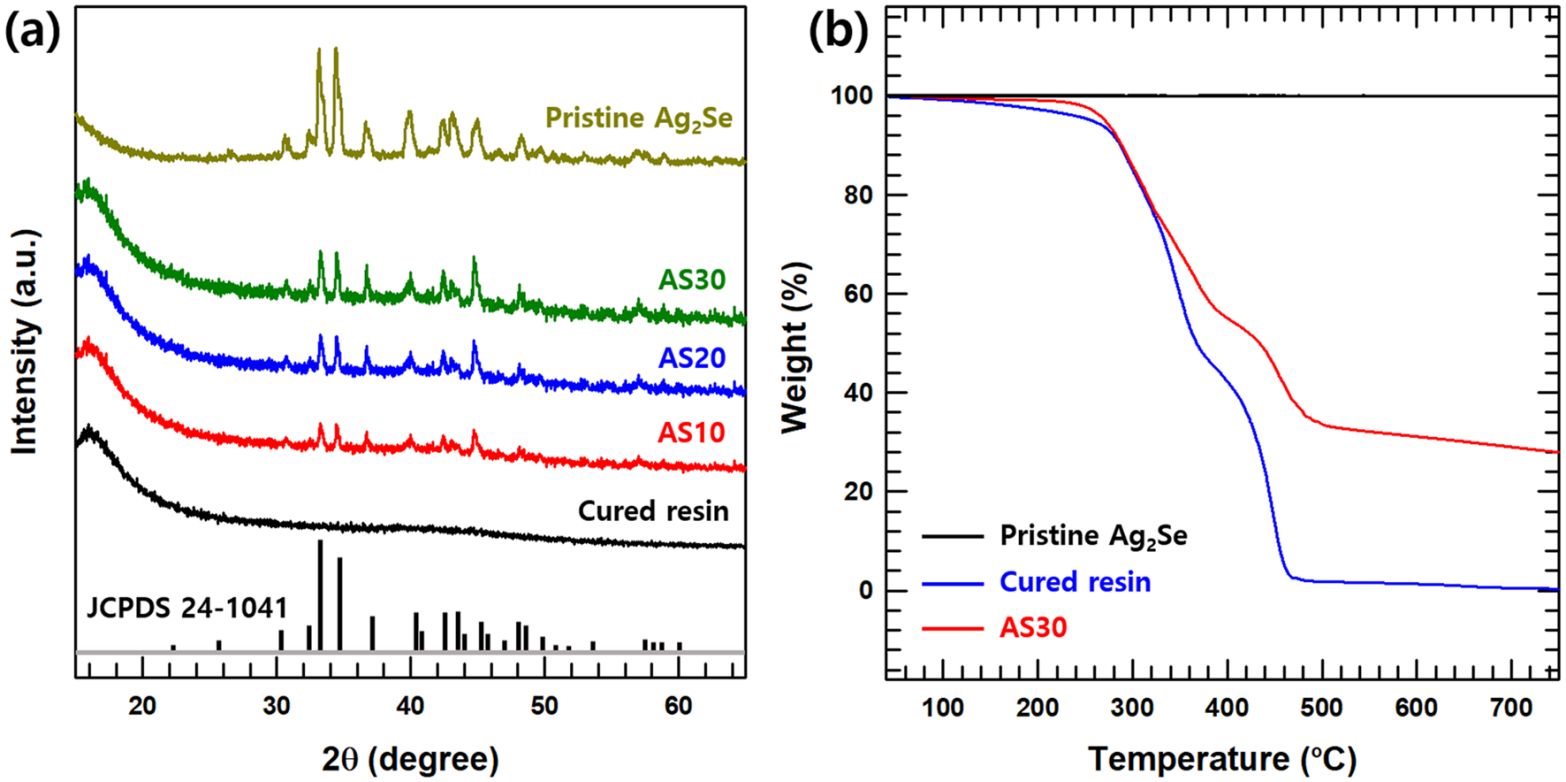
(**a**) XRD patterns of cured resin, AS10, AS20, AS30, and pristine Ag_2_Se. (**b**) TGA curves for pristine Ag_2_Se, cured resin and AS30 under N_2_ atmosphere.

XRD analysis was conducted to confirm the curing of the composite resin samples and to analyze the crystalline structure. The XRD patterns of the pristine Ag_2_Se NW and printed sample, with various Ag_2_Se contents (0, 10, 20, and 30 wt.%), are shown in Fig. 2a. The peaks in the XRD patterns of Ag_2_Se can be indexed to the orthorhombic phase of Ag_2_Se (JCPDS no. 24-1041). These results confirm that pristine Ag_2_Se was successfully synthesized. For the printed composite samples, it was found that the peaks related Ag_2_Se increased as the content of Ag_2_Se increases. However, since the intensity of the pristine photoresin peak is relatively weaker than that of Ag_2_Se, it is difficult to meaningful confirm the change of the peak according to the contents of the fillers.

The thermal degradation behavior of an Ag_2_Se, cured photoresin, and cured composite resin (AS30) is shown in Fig. 2b. A TGA was performed under N_2_ gas to estimate the composition of the cured composites. The pristine Ag_2_Se showed thermal stability up to ~ 800 °C. However, the weight loss of the cured photoresin appeared around ~ 270 °C and ~ 380 °C. This characteristic is believed to be due to the different temperatures at which the masses of the two materials (DUDMA and IBOA) were used in photoresin. The TGA curve of two pristine materials with BAPO is shown in Fig. S2. The two weight loss points of the photoresin coincide with the weight loss point of each material. From these TGA data, the weight fraction of Ag_2_Se was confirmed (~ 28 wt.%).

FE-SEM analysis was conducted to characterize the morphology of the cured sample. The FE-SEM images of Ag_2_Se NW are shown in Fig. S3a. The images in Fig. S3a highlight the presence of distributed wire structures. The average length and diameter of Ag_2_Se are ~ 20 nm and ~ 10 μm. After Ag_2_Se mixed with the photoresin and photoinitiator, the mixture was then printed into a various sample.

**Figure 3:**
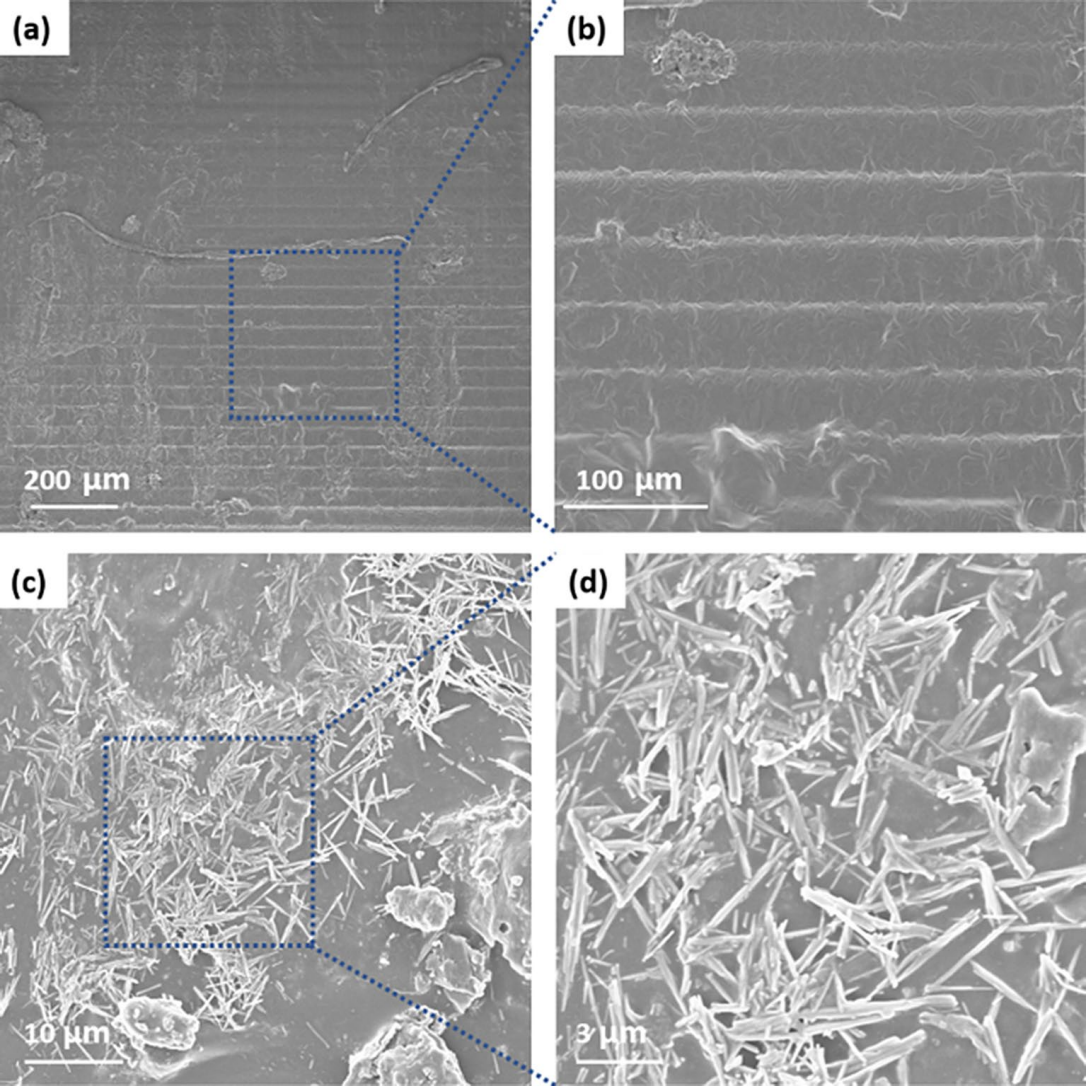
(**a**) Low and (**b**) high-magnification cross sectional FE-SEM images of cured resin. (**b**) low and (**c**) high-magnification cross sectional FE-SEM images of AS30 composite sample.

Figure 3a,b shows the morphology cured photoresin. This image confirmed that the layered structure of a sample was cured by the DLP 3D printer. The low-and high magnification FE-SEM images of the AS30 sample are illustrated in Fig. 3c,d. The FE-SEM image (Fig. 3c,d) demonstrates the well-dispersion of Ag_2_Se, showing the morphology of the Ag_2_Se NW similar to Fig S2. Thus, the AS30 hybrid composite was successfully fabricated with a resin mixing and 3D printing process. Also, the surface FE-SEM images of AS30 are shown in Figs. S4a-b. In these figures, the dispersion of Ag_2_Se fillers is clearly shown.

**Figure 4:**
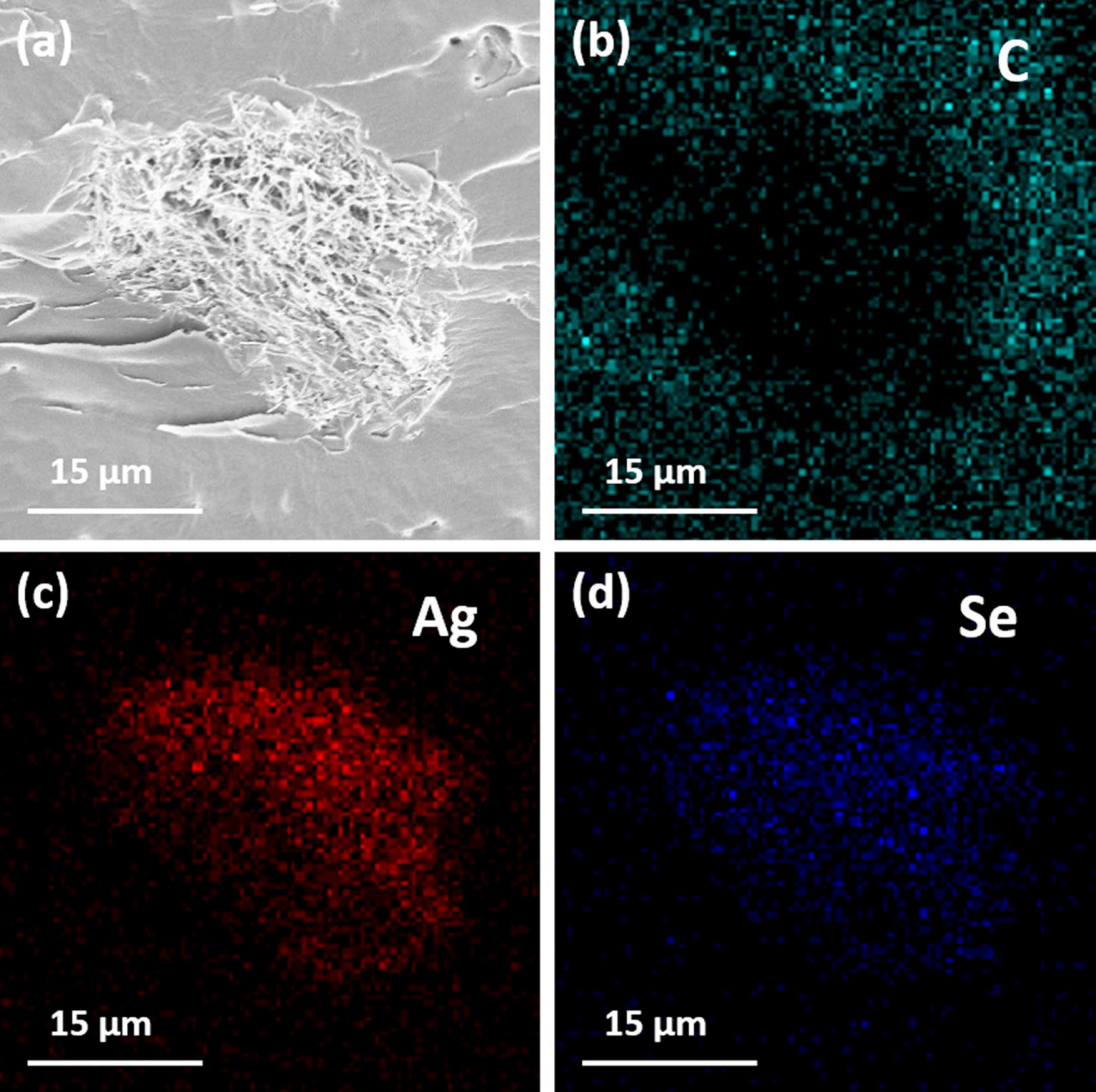
(**a**) Cross sectional FE-SEM images of AS30, and the corresponding EDS elemental mappings of (**b**) C, (**c**) Ag, and (**d**) Se atoms.

The successful curing of resins containing an Ag_2_Se filler is in good agreement with the results found in the EDS analysis. Figure 4a–d shows the FE-SEM images and corresponding EDS mappings of the AS30 composite sample. No visible images other than Ag_2_Se were detected in the filler part of the composite, due to the strong signal of the Ag_2_Se filler compared to the composite resin. However, in other parts of the resin, image of C was relatively strong.

**Figure 5:**
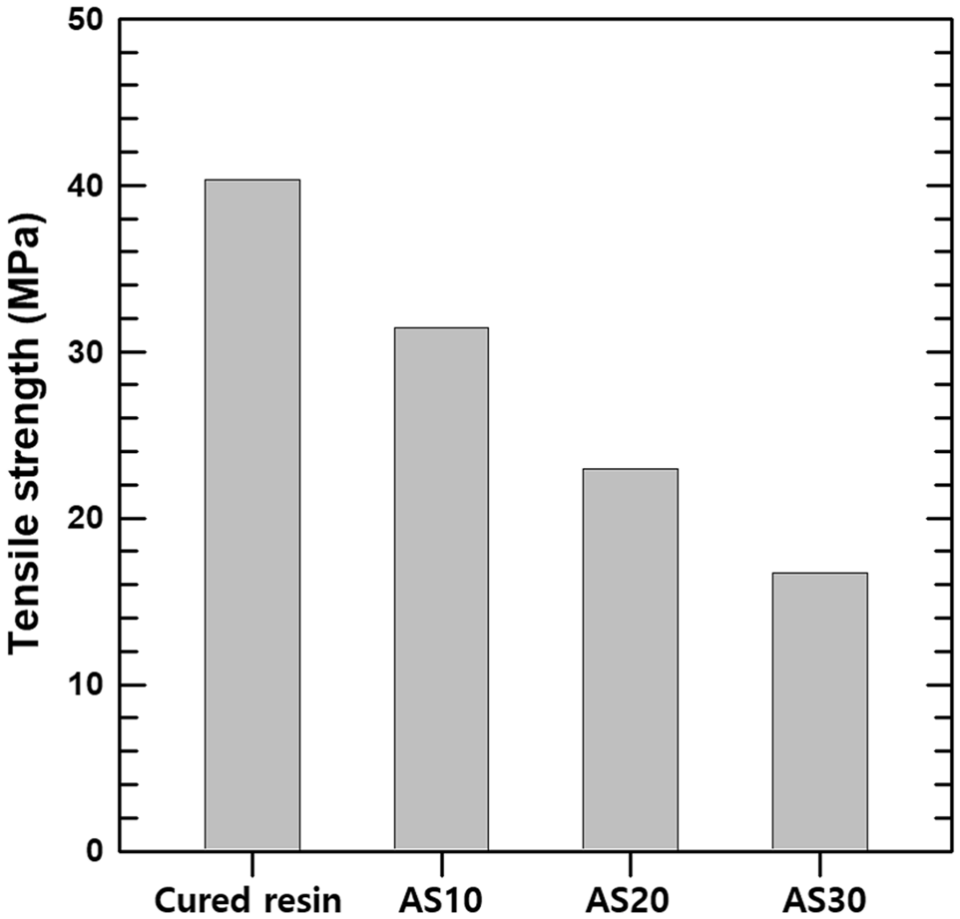
Tensile strength of various dog-bone shaped composite measured by UTM.

The mechanical properties of AS10, AS20, AS30, and the cured resin were studied. The dog-bone-shaped composite (Fig. S1) were used to analyze the mechanical properties of composite. The tensile strength of the various composite samples is plotted in Fig. 5. The tensile strength of the photoresin, AS10, AS20, and AS30 composite is 40.3, 31.5, 23.0, and 16.7 MPa. These mechanical properties allow the composites to withstand harsh mechanical environments.

For TE property measurement, the composites were printed with disks, which have 1 mm thickness and 25.4 mm diameter. Pristine Ag_2_Se samples were also prepared with a disk sample of the same size, which was prepared with hot-press method.

**Figure 6:**
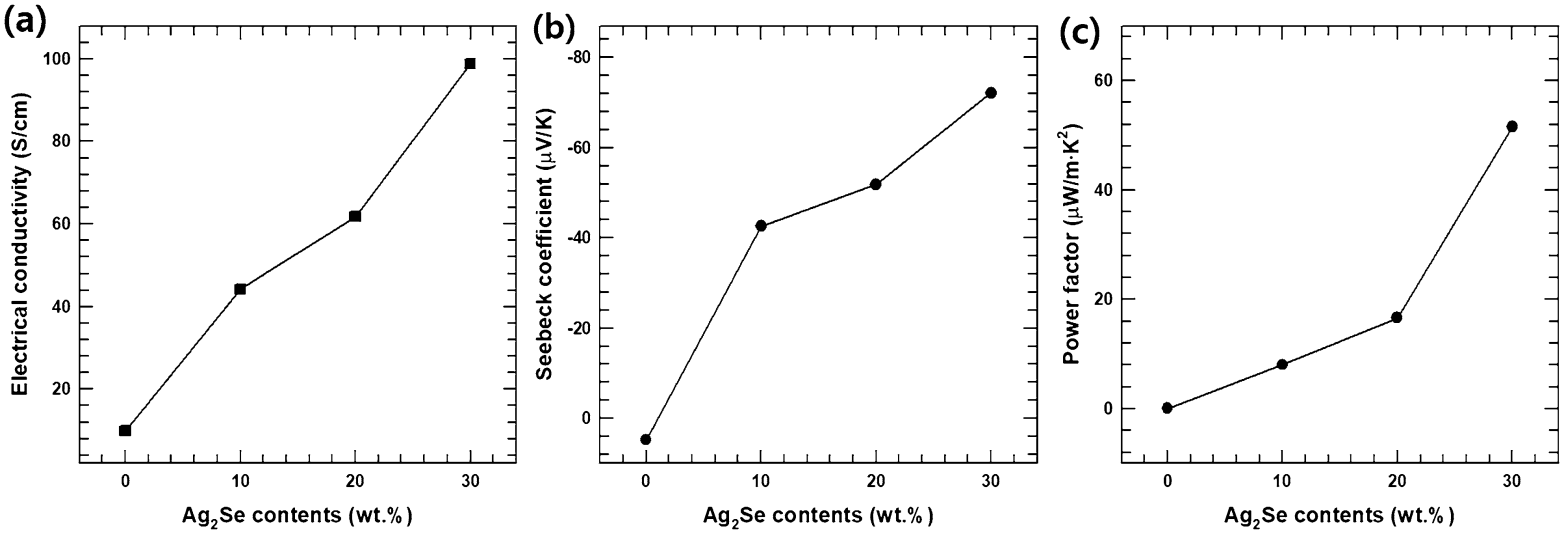
(**a**) Electrical conductivity, (**b**) Seebeck coefficient, and (**c**) power factor of composite sample with various AS contents.

The room-temperature electrical conductivity of 4 composite samples (cured photoresin, AS10, AS20, and AS30) was shown in Fig. 6a. The electrical conductivity of pristine Ag_2_Se is ~ 680 S/cm, which was similar to the previous reports which analyzed the electrical conductivity of Ag_2_Se^30,31^. The composite’s electrical conductivity was increased with 44.3 S/m to 98.9 S/cm with an increase of Ag_2_Se contents from 10 to 30 wt.%. This increase in electrical conductivity is because of the relatively higher electrical conductivity of pristine Ag_2_Se.

Figure 6b shows the Seebeck coefficient of 4 composite samples at room temperature. Similar to the electrical conductivity, the absolute value of Seebeck coefficient also tends to increase with increasing Ag_2_Se content. This trend is also caused by the difference in the Seebeck coefficient of the filler and resin. The Seebeck coefficient of Ag_2_Se is ~ − 145 μV/K, which is similar to previous studies^32,33^. Also, the Seebeck coefficients of AS10, AS20, and AS30 are negative values, which indicates that the composite materials exhibit an N-type electrical transport behavior. Thus, most of the charge carriers in the composite are electrons.

The difference in electrical conductivity and Seebeck coefficient is further confirmed by the following relations.

$$\sigma=n \cdot e \cdot \mu$$

$$\mathrm{S}= \frac{8\cdot {\pi }^{2}\cdot {{k}_{B}}^{2}}{3\cdot e\cdot {h}^{2}}\cdot {m}^{*}\cdot T\cdot {\left(\frac{\pi }{3\cdot n }\right)}^\frac{2}{3}$$

where *n*, *e*, *μ*, *k*_*B*_*, h,* and m* are the carrier concentration, electron charge, carrier mobility, Boltzmann constant, Planck constant, and effective mass of the carrier, respectively. The carrier concentration and carrier mobility of composite samples are shown at Fig. S5. As the contents of Ag_2_Se increases, the carrier conductivity tends to decrease, and the carrier mobility shows the increasing trend.

The room-temperature power factor (*PF* = *S*^*2*^* · σ*) of the 4 composite samples is illustrated in Fig. 6c. The maximum power factor of the composites is ~ 51.5 μW/m·K^2^ at room temperature, which is seen in the AS30 sample. This increase is because of the increase of the Seebeck coefficient and electrical conductivities of the composite sample.

**Figure 7:**
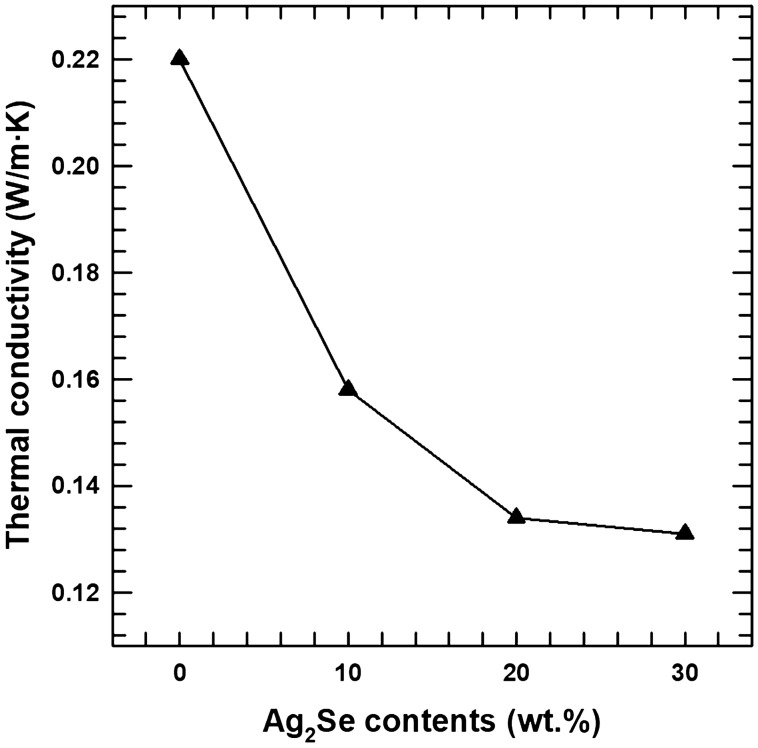
Thermal conductivity of composite sample with various AS contents.

The total thermal conductivities of composite samples are shown in Fig. 7. The total thermal conductivity of the cured resin was 0.22 at room temperature, and the thermal conductivity of the AS10, AS20, and AS30 composite samples shows a lower value than that of the pristine resin. The thermal conductivity is composed of two terms, which are the electrical term (*κ*_*e*_) and the lattice term (*κ*_*l*_) (*κ* = *κ*_*e*_ + *κ*_*l*_). The electronic thermal conductivity can be estimated from the Wiedemann–Franz law; *к*_*e*_ = *L · T · σ,* where L is the Lorentz number (L = 2.44 × 10^–8^ V^2^/K^2^)^34,35^. The total thermal conductivity is hardly affected by the electronic contribution due to its low electrical conductivity. Thus it mainly depends on the lattice term and the lattice thermal conductivity which, in turn, are mainly dependent on lattice phonon scattering. The composite sample contains Ag_2_Se, while the fillers were introduced into uncured photoresin. This addition makes more heterointerfaces between different materials. These boundaries generated from different materials increase phonon scattering, leading to a decrease in *κ*_*l*_. The results on the phonon scattering effect of different interfaces between these composite materials are supported by previous study^36^.

**Figure 8:**
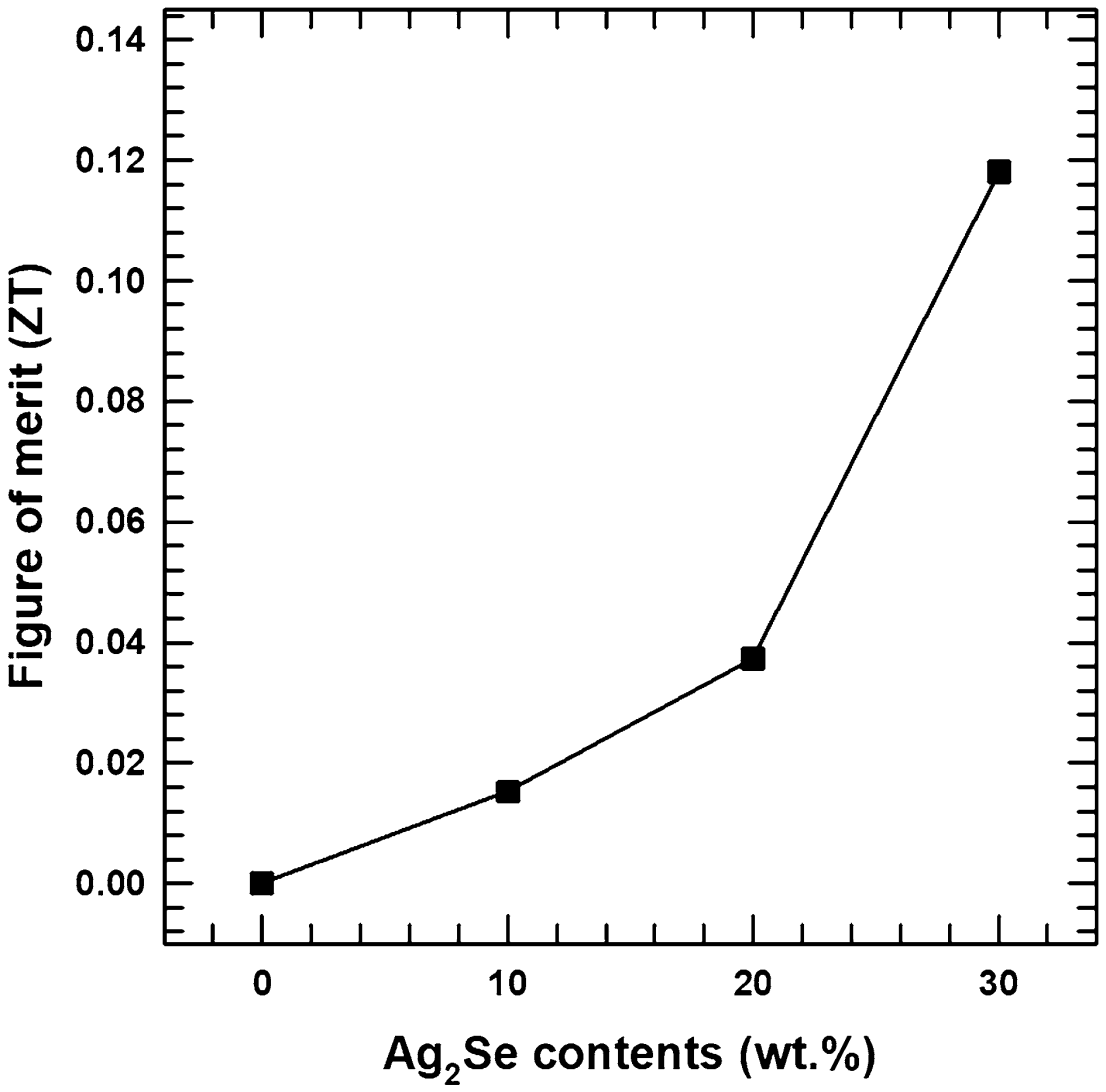
ZT values of composite sample with various AS contents.

Based on this measured power factor and thermal conductivity, the thermoelectric figure of merits (*ZT* = *S*^*2*^* · σ · T / к*) was calculated, and these values are shown in Fig. 8. These ZT values show an increasing tendency with increasing Ag_2_Se contents. The AS30 composite sample shows a maximum ZT value which is ~ 0.12 at room temperature. Additionally, temperature-dependent thermoelectric properties of composite samples are shown in Fig. S6 and S7. The electrical conductivity is seen to gradually increases with increasing temperature, while the Seebeck coefficient initially increase until a temperature of ~ 450 K is reached, after thatm it subsequently decreases with further increase in temperature. This tendency is similar to other previous studies using 3D-printed thermoelectric materials^37^. In this work, we demonstrated the technology of manufacturing thermoelectric materials through a 3D printing technique. Photoresins containing Ag_2_Se were successfully synthesized and cured with a 3D printer. The thermoelectric properties of the composites are lower than that of the pristine Ag_2_Se, but the use of 3D printing technology enables the creation of various shapes of thermoelectric materials that are difficult to obtain with conventional methods, which can greatly expand the field of thermoelectric applications.

## Conclusions

The purpose of this study is to fabricate a thermoelectric composite with improved mechanically and thermoelectric properties. For this purpose, we synthesized the UV-curable resin containing DUDMA and IBOA because of their low viscosity and high miscibility. These composite resins showed high mechanical strength. Then, various contents of AS fillers were added to the composite resins and cured. The maximum filler content is 30 wt.% of AS. However, due to the light penetration issue during 3D printing and high viscosity, filler loading over 30 wt.% could not be processed. After curing, the composite’s microstructure and morphology were analyzed with XRD, FE-SEM, and EDS analysis. Through these analyses, it was confirmed that the composite resin retains its composition after curing. The mechanical and thermoelectric properties were measured after successful printing. With increasing filler contents, the Seebeck coefficient and electrical conductivity of the printed sample showed an increasing trend. Composite sample with 30 wt.% of AS shows the maximum power factor of ~ 51.5 μW/m·K^2^ at 300 K after curing. The results of this study show that thermoelectric composites can easily be synthesized using 3D printing. We also believe that expanding these approaches will allow the versatility of 3D printing to be applied to the production of electronic and energy materials.

## Supplementary Information

Supplementary Information.

## Data Availability

All data generated or analyzed during this study are included in this paper. Raw datasets are available from the corresponding author on reasonable request.
