# Supplementary material for: Enhanced thermoelectric performance of UV-curable silver (I) selenide-based composite for energy harvesting
Source: Sci Rep. 2021 Aug 17;11:16683. doi: 10.1038/s41598-021-96267-x (PMC8371107; doi:10.1038/s41598-021-96267-x)
Supplement: Supplementary file 1 — Supplementary Information. [file 41598_2021_96267_MOESM1_ESM.docx]

**Supplementary Information:**

**Enhanced thermoelectric performance of UV-curable silver (I) selenide-based composite for energy harvesting.**

Dabin Park^a^, Seonmin Lee^a^ and Jooheon Kim^a,b,c^*

^a^School of Chemical Engineering & Materials Science,

Chung-Ang University, Seoul 06974, Republic of Korea

^b^Department of Advance Materials Engineering,

Chung-Ang University, Anseong 17546, Republic of Korea

^c^Department of Intelligent Energy and Industry, Graduate School,

Chung-Ang University, Seoul 06974, Republic of Korea

*Corresponding author: jooheonkim@cau.ac.kr (J. Kim)

Supporting Information Contents:

1. Supplementary Notes

2. Figures

3. Tables

**Note 1. Expeimental Methods**

***1.1. Materials***

Diurethane dimethacrylate (DUDMA), Isobornyl acrylate (IBOA), and Phenylbis(2,4,6-trimethylbenzoyl) phosphine oxide (BAPO) were purchased from Sigma-Aldrich (USA). Selenium dioxide (SeO_2_), L-ascorbic acid, *β*-cyclodextrin (*β*-CD), ethylene glycol (EG), silver nitride (AgNO_3_), polybinylpyrrolidone K30 (PVP), and ethanol (C_2_H_5_OH) were purchased from Daejung Chemical & Materials Co. (Seoul, Korea). All chemicals were used without further purification.

***1.2. Ag_2_Se NW preparation***

This experimental method is same as that performed in the previous paper.^1^ SeO_2_ (1 g) and *β*-CD (1 g) were added to 100mL of DI water under vigorous stirring to produce solution I. In a separate beaker, solution II was prepared with 1 g of ascorbic acid with 100 mL of DI water. After solution II was completely dissolved, the solution was poured into the solution I. The color of the mixture became brick-red, indicating the formation of Se colloids. After a reaction time of 4 h, the mixture was then centrifuged and washed with DI water and ethanol several times. The synthesized products were then dispersed in ethanol for 12 h, allowing time for a Se NWs to form. Then, the final Se NWs were collected by drying in a vacuum oven at 80 °C overnight.

The Ag precursor solution was prepared with 1 g of AgNO_3_ and 100 mL of EG. This Ag precursor solution was then poured into the Se NW solution. The as-synthesized Se NW solution was prepared with 0.2 g of as-prepared Se NWs and 1 g of PVP K30 dissolved in 100 mL of EG in another. After stirring for 2h, the Ag_2_Se NW solution was then centrifuged and washed with DI water and ethanol several times. Finally, the synthesized Ag2Se NWs were dried in a vacuum oven at 80 °C overnight.

***1.3. Photoresin formulations***

All composite resins were stirred in the light-sealed condition until a homogeneous composite was formed. DUDMA, IBOA, and BAPO were used as a monomer, crosslinker, and photoinitiator. The ratio of monomer and crosslinker is 1:1. Thereafter, Ag_2_Se powders were dispersed in uncured resin with various contents (10, 20, and 30 wt.%). Also, immediately before curing, a photoinitiator of about 0.5 wt.% of the resin was added. The amounts and composition of each composite resins are shown in Table S1.

***1.4. Composite preparation***

A DLP printer (ASIGA Max) was used to create a 3D-Ag_2_Se composite sample through a curing process, with a light intensity of 37.49 mW/cm^2^ and exposure time of 5 s for each layer (0.05 mm thickness). After the curing process, all samples were thermally annealed in N_2_ in a tubular furnace at 400 °C, for 5 h.

***1.5. Characterization***

X-ray diffraction (XRD; New D8-Advance/Bruker-AXS) at 40 mA, 40 kV using a Cu-K*α* radiation (0.154056 nm) source, and a scan rate of 1°·s^-1^ in the 2θ range of 5–70° was employed to characterize the crystal structure of the materials. The thermal degradation of the composites was examined by thermogravimetric analysis (TGA) (TGA-2050, TA instruments) at a heating rate of 10 ℃/min and a temperature range of RT to 800 ℃, under an N_2_ atmosphere. The microstructure and morphology of the composite sample were evaluated using Field-emission scanning electron microscopy. The elemental mappings of the composites were analyzed by energy-dispersive X-ray spectroscopy (EDS, NORAN system 7, Thermo Scientific). The mechanical properties were measured using a universal testing machine (UTM, 3344Q9465, Instron Co.) at a crosshead speed rate of 5 mm/min. The electrical conductivity of the composites was measured by a four-point probe method (Keithley 2400 Source Meter). A homemade device containing a pair of voltmeter and thermocouples was used to measure the Seebeck coefficient, according to the linear relationship (*S* = *ΔV*/*ΔT*). The thermal conductivity of the composites was calculated from the relation *κ = C_p_·α·ρ*, where *C_p_*, *α*, and *ρ* are the specific heat, thermal diffusivity, and bulk density of the composite, respectively. The laser flash analysis (LFA, Netzsch Instruments Co., NanoFlash LFA 467) method was employed to measure the room-temperature thermal diffusivity. Charge carrier concentration and carrier mobility of the composite were determined by conducting Hall-effect measurements using a Van der Pauw four-point probe configuration (HMS-3000, Ecopia).

**2. Figures**

**
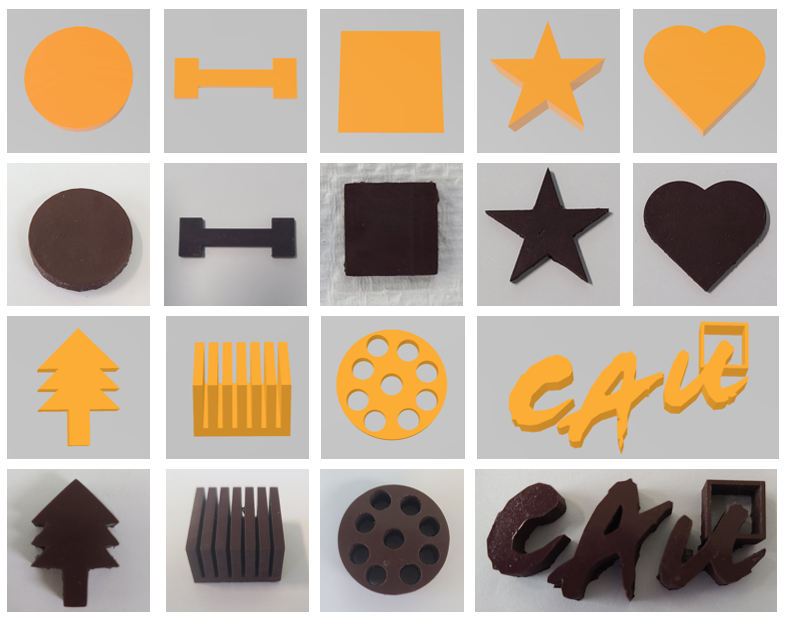
**

**Figure S1.** CAD design and photograph of a printed composite sample with various shape.


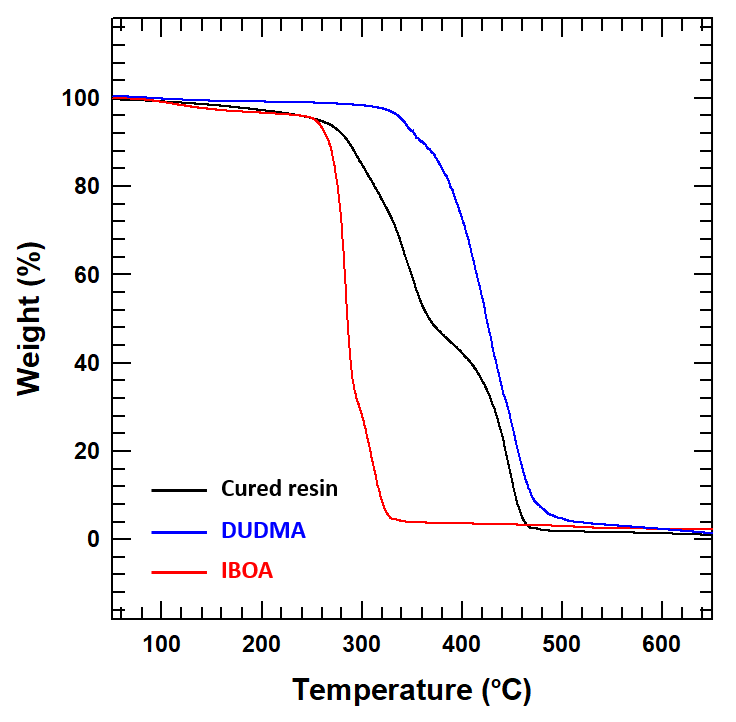


**Figure S2.** TGA curve for cured resin, DUDMA resin, and IBOA resin.


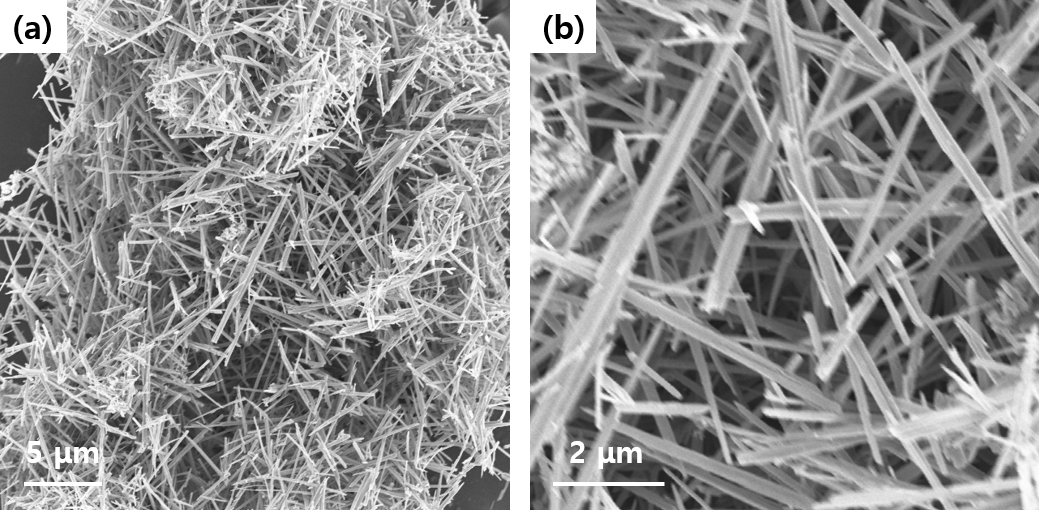


**Figure S3.** (a) Low and (b) high-magnification FE-SEM images of Ag_2_Se NWs.


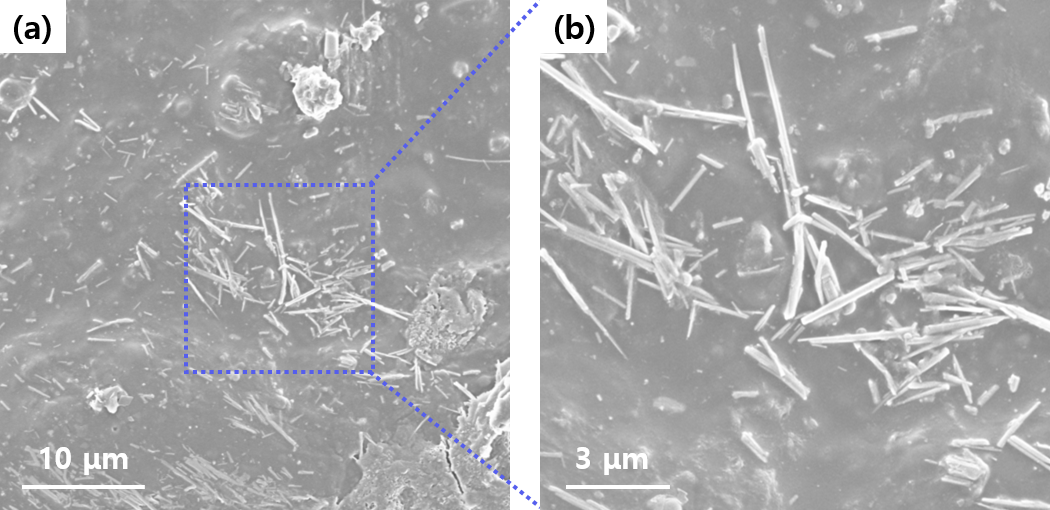


**Figure S4.** (a) Low and (b) high-magnification surface FE-SEM images of AS30 composite sample.


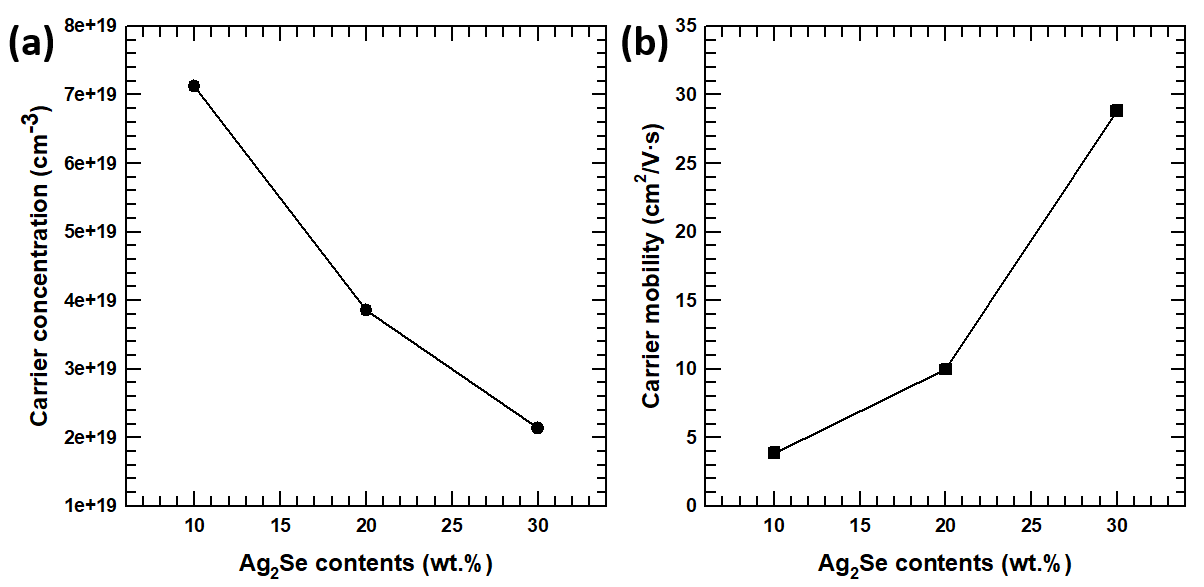


**Figure. S5.** (a) Carrier mobility, and (b) carrier concentration of of various composite samples.

**
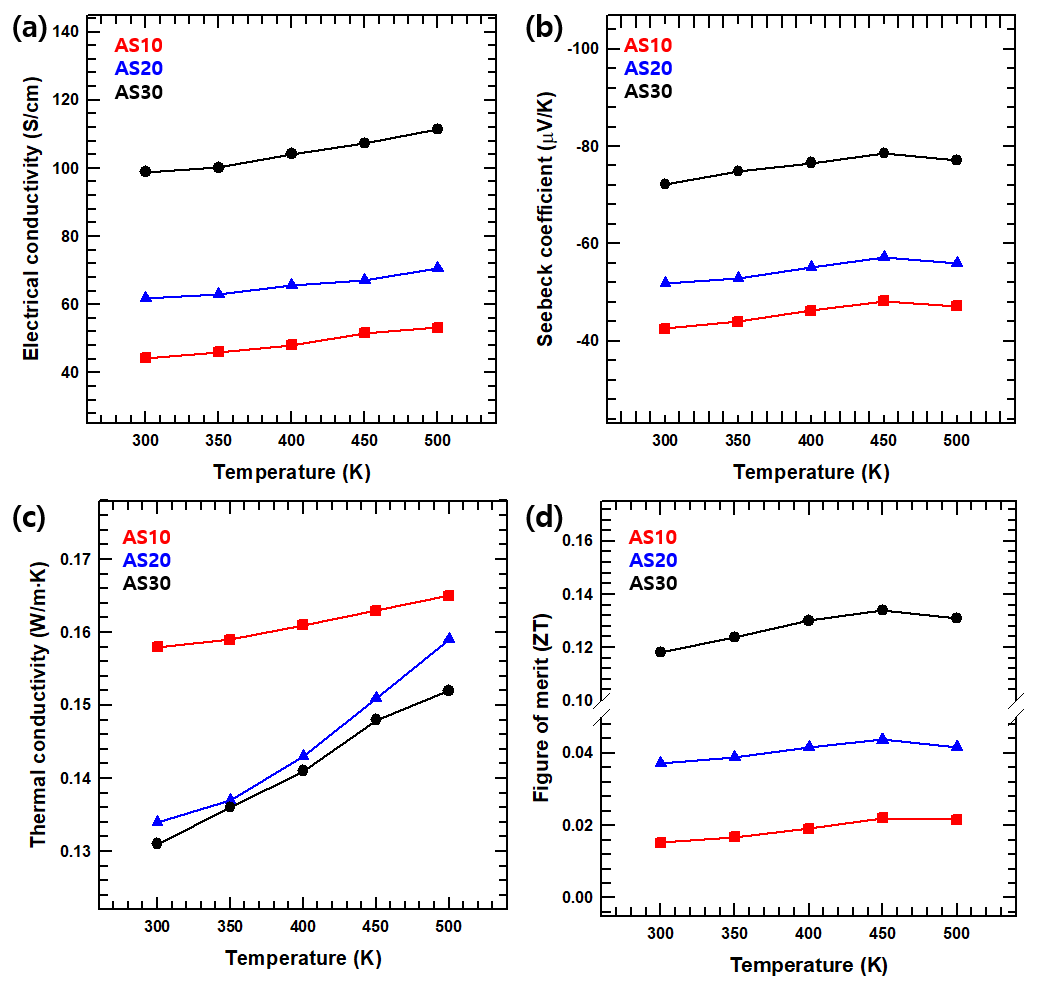
 Figure S6.** Temperature-dependent (a) electrical conductivity, (b) Seebeck coefficient, (c) thermal conductivity, and (d) ZT value of various composite samples.


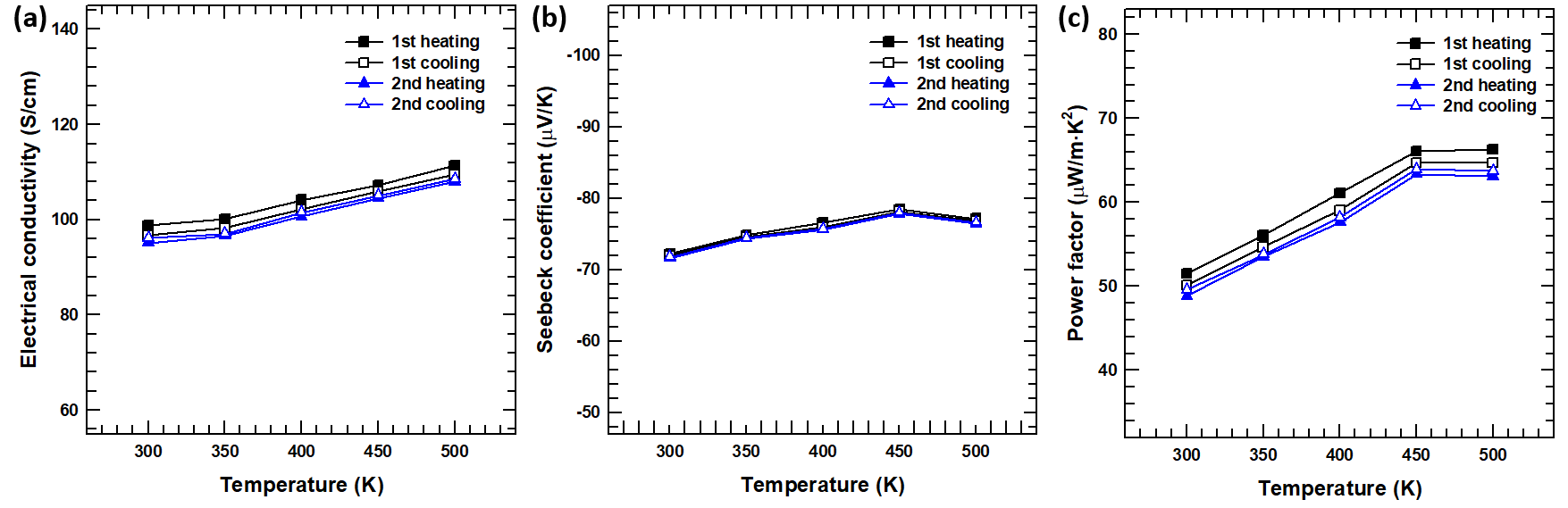


**Figure S7.** Temperature dependent (a) electrical conductivity, (b) Seebeck coefficient, and (c) power factor values of AS30 composite during multiple heating and cooling systems.

**3. Tables**

| Resin | Materials | | | Filler composition (%) |
| --- | --- | --- | --- | --- |
|  | DUDMA (g) | IBOA (g) | Ag_2_Se (g) |  |
| Photoresin | 15 | 15 | - | 0 |
| AS10 | 13.5 | 13.5 | 3 | 10 |
| AS20 | 12 | 12 | 6 | 20 |
| AS30 | 10.5 | 10.5 | 9 | 30 |

**Table S1.** Experimental condition for preparation of composites with various ratios of AS fillers.

**References**

1 Park, D., Ju, H. & Kim, J. Enhanced thermoelectric properties of flexible N-type Ag_2_Se nanowire/polyvinylidene fluoride composite films synthesized via solution mixing. *J. Ind. Eng. Chem.* **93**, 333-338 (2021).
